# Safety, immunogenicity, and effectiveness of COVID-19 vaccines for pregnant persons: A protocol for systematic review and meta analysis

**DOI:** 10.1097/MD.0000000000032954

**Published:** 2023-03-03

**Authors:** Agustín Ciapponi, Mabel Berrueta, Jamile Ballivian, Ariel Bardach, Agustina Mazzoni, Steven Anderson, Fernando J. Argento, Karin Bok, Daniel Comandé, Erin Goucher, Beate Kampmann, Edward P. K. Parker, Federico Rodriguez-Cairoli, Victoria Santa Maria, Andy Stergachis, Gerald Voss, Xu Xiong, Sabra Zaraa, Flor M. Munoz, Ruth A. Karron, Sami L. Gottlieb, Pierre M. Buekens

**Affiliations:** a Instituto de Efectividad Clínica y Sanitaria (IECS-CONICET), Buenos Aires, Argentina; b US Food and Drug Administration, CBER, WA, Columbia; c National Institute of Allergy and Infectious Diseases (NIAID), Vaccine Research Center, Bethesda, MD; d School of Public Health and Tropical Medicine, Tulane University, New Orleans; e The Vaccine Centre, London School of Hygiene and Tropical Medicine, London WC1E 7HT, UK; f Vaccines & Immunity Theme, MRC Unit The Gambia at the London School of Hygiene and Tropical Medicine, Banjul, Gambia; g School of Pharmacy and School of Public Health, University of Washington, Seattle, WA; h Coalition for Epidemic Preparedness Innovations, Oslo, Norway; i Baylor College of Medicine, Texas Children’s Hospital, Houston, TX; j Center for Immunization Research, Bloomberg School of Public Health, Johns Hopkins University, Baltimore, MD; k Medical Officer, Department of Sexual and Reproductive Health and Research, World Health Organization, Geneva, Switzerland.

**Keywords:** COVID-19, meta-analysis, pregnancy, protocol, systematic review, vaccine

## Abstract

Numerous vaccines have been evaluated and approved for coronavirus disease 2019 (COVID-19). Since pregnant persons have been excluded from most clinical trials of COVID-19 vaccines, sufficient data regarding the safety of these vaccines for the pregnant person and their fetus have rarely been available at the time of product licensure. However, as COVID-19 vaccines have been deployed, data on the safety, reactogenicity, immunogenicity, and efficacy of COVID-19 vaccines for pregnant persons and neonates are becoming increasingly available. A living systematic review and meta-analysis of the safety and effectiveness of COVID-19 vaccines for pregnant persons and newborns could provide the information necessary to help guide vaccine policy decisions.

**Methods and analysis:**

We aim to conduct a living systematic review and meta-analysis based on biweekly searches of medical databases (e.g., MEDLINE, EMBASE, CENTRAL) and clinical trial registries to systematically identify relevant studies of COVID-19 vaccines for pregnant persons. Pairs of reviewers will independently select, extract data, and conduct risk of bias assessments. We will include randomized clinical trials, quasi-experimental studies, cohort, case-control, cross-sectional studies, and case reports. Primary outcomes will be the safety, efficacy, and effectiveness of COVID-19 vaccines in pregnant persons, including neonatal outcomes. Secondary outcomes will be immunogenicity and reactogenicity. We will conduct paired meta-analyses, including prespecified subgroup and sensitivity analyses. We will use the grading of recommendations assessment, development, and evaluation approach to evaluate the certainty of evidence.

## 1. Introduction

### 1.1. The burden of coronavirus disease 2019 (COVID-19) disease in pregnant persons

In November 2019, a novel coronavirus named severe acute respiratory syndrome coronavirus 2 (SARS-CoV-2) was described for the first time in Wuhan, China. Its spread led to a global outbreak of the respiratory condition named COVID-19.^[1,2]^ In March 2020, the World Health Organization (WHO) declared the COVID-19 pandemic.^[3]^ Studies have shown that pregnant persons with COVID-19 are at increased risk of severe illness compared to nonpregnant persons and exhibit a higher risk of adverse pregnancy- and birth outcomes.^[4–7]^ Although pregnant persons with COVID-19 diagnosed in the hospital are less likely to present with or manifest symptoms of fever, dyspnea, and myalgia than nonpregnant persons of childbearing age, they are at increased risk of intensive care unit admission, need for invasive ventilation, and treatment with extracorporeal membrane oxygenation.^[4]^ Increasing maternal age, high body mass index, nonwhite ethnicity, preexisting comorbidities, gestational diabetes mellitus, and preeclampsia are risk factors for severe COVID-19 during pregnancy.^[4,7]^ Pregnant persons with COVID-19 are also at higher risk of experiencing preterm birth, and their neonates are more likely to require neonatal intensive care unit admission.^[4,7]^

### 1.2. Evidence of the effects of COVID-19 vaccines in pregnant persons

Pivotal efficacy trials of COVID-19 vaccines excluded pregnant persons from their eligible population.^[8,9]^ Multiple vaccine products, therefore, had limited human data on their safety during pregnancy at the time of their approval for widespread use. Nonetheless, a growing number of regulatory bodies have either permitted or recommended the use of COVID-19 vaccines in pregnant persons on the basis that the benefits of vaccination are likely to outweigh the potential risks. Consequently, more studies are beginning to report the effects of COVID-19 vaccines in pregnant persons.^[10–17]^ Most published studies include data only for mRNA vaccines, such as BNT162b2 and mRNA-1273, primarily from high-income countries.^[18,19]^ There is still an urgent need for information regarding other COVID-19 vaccines that have been widely distributed in low and middle-income countries, such as SPUTNIK-V (Gam-Covid-Vac), BBIBP-CorV (Sinopharm), CoronaVac (Sinovac), ChAdOx1 (Vaxzevria; also known as AZD1222 or Covishield) and Ad26.COV2.S (Janssen-Cilag/Johnson and Johnson).

With the continuous and rapid growth of data, a living systematic review (LSR) and living meta-analysis that continuously collects and assesses the latest research findings as they become available is probably the most suitable method to disseminate the up-to-date evidence in a timely fashion to assist decision-making. This LSR and living meta-analyses aim to provide an up-to-date synthesis of available evidence to support evidence-based recommendations regarding the benefits and potential harms of COVID-19 vaccines for pregnant persons.

## 2. Methods

This protocol is reported according to the guideline provided in the preferred reporting items for systematic reviews and meta-analysis protocols statement (See table Supplemental Digital Content 1, http://links.lww.com/MD/I458, which presents preferred reporting items for systematic reviews and meta-analysis protocols checklist for this protocol).^[20]^ It was registered in the International Prospective Register of Systematic Reviews (PROSPERO CRD42021281290) database. The review will follow recommendations outlined in The Cochrane Handbook of Systematic Review of Interventions^[21]^ and PRISMA.^[22,23]^

### 2.1. Inclusion criteria for considering studies for this review

Types of studies: We will include randomized clinical trials, quasi experimental studies, and observational studies irrespective of publication status, publication year, and language. We will also include case reports for rare adverse events.

### 2.2. Literature search strategy (see document Supplemental Digital Content 2, http://links.lww.com/MD/I459, which provides the search strategies)

• Biweekly, we will systematically search published and unpublished studies, without restrictions on language or publication status, from January 2020 in order to incorporate relevant new evidence as it becomes available.^[24]^ An experienced librarian will search the Cochrane Library databases, MEDLINE, EMBASE, latin american and caribbean health sciences literature, Science Citation Index Expanded, China Network Knowledge Information, Chinese Biomedical Literature Database, Chinese Science Journal Database, WHO Database of publications on SARS-CoV-2, EPPI-Centre map of the current evidence on COVID-19, guidelines published by national and international professional societies (e.g., American College of Obstetricians and Gynecologists, Royal College of Obstetricians and Gynecologists, International Federation of Gynecology and Obstetrics), preprint servers (ArXiv, BiorXiv, medRxiv, search.bioPreprint), and COVID-19 research websites (Global research on COVID-19 supported by the WHO, COVID-19 Vaccine Tracker, the L-OVE Platform, and the COVID-19 Living Evidence.
• We will contact experts in the field relevant to our review question. We will hand search the reference lists of the identified systematic reviews to identify relevant studies missed by our search strategy.

### 2.3. Types of participants and sample size

Study participants are pregnant persons, irrespective of prior exposure to COVID-19, age, comorbidities, immune status, risk group, and their newborns. We will include observational studies reporting safety outcomes with sample sizes of at least 50 subjects and immunogenicity studies with samples of at least 10 subjects. Case reports of infrequent adverse events will be included, regardless of sample size.

### 2.4. Types of interventions

#### 1.2.4. Intervention/exposure.

COVID-19 vaccines authorized by WHO and/or authorized or approved by any national regulatory authority, irrespective of doses and administration schedule.

#### 2.2.4. Comparator.

Any control group, including usual care, no intervention, another COVID-19 vaccine, or any other “active” comparator regardless of co-interventions used (i.e., flu vaccine).

We will also include noncomparative studies; therefore, a control group will not be mandatory for these outcomes.

### 2.5. Primary outcomes

1. Safety outcomes: We will use the 21 standardized case definitions developed by global alignment of immunization safety assessment in pregnancy of prioritized obstetric and neonatal outcomes based on the standard Brighton Collaboration process.^[25]^
  a. *Obstetric outcomes:* Hypertensive disorders of pregnancy, maternal death, non-reassuring fetal status, pathways resulting in preterm birth and postpartum hemorrhage, abortion/miscarriage, antenatal bleeding, gestational diabetes, dysfunctional labor, fetal growth retardation.
  b. *Neonatal outcomes:* Congenital anomalies, neonatal death, neonatal infections, preterm birth, stillbirth, low birth weight, small for gestational age, neonatal encephalopathy, respiratory distress, failure to thrive, and microcephaly.
2. Vaccine efficacy/effectiveness outcomes
  a. Prevention of confirmed and symptomatic mild/moderate/severe COVID-19 by nucleic acid amplification tests, such as real-time polymerase chain reaction, with or without serological or virological evidence of past SARS-CoV-2 infection.
  b. Prevention of complications attributed to COVID-19 (including hospital-attended COVID-19 and death).
  c. All-cause mortality.
3. Adverse events following immunization:
  a. Serious adverse events: any serious adverse events due to vaccine administration
4. Immunogenicity measurements: cellular and humoral immune responses measurements

### 2.6. Secondary outcomes

1. Safety outcomes:
  a. Maternal and neonatal outcomes not specified by the global alignment of immunization safety assessment in pregnancy definitions.
2. Vaccine efficacy/effectiveness outcomes
  a. Prevention of asymptomatic SARS-CoV-2 infection: determined by asymptomatic seroconversion of the N-binding antibody and/or asymptomatic SARS-CoV-2 infection based on central laboratory confirmed nucleic acid amplification tests, such as real time polymerase chain reaction.
  b. Prevention of mother-to-child transmission: Presence and persistence of SARS-CoV-2 (viral load, protective antibodies, RBD Antigen-specific ELISA (IgG), Spike (S) antibody, Neutralization of live SARS-CoV-2, Neutralization of a pseudovirus modified to express SARS-CoV-2 antigens, SARS-CoV-2 antibody -IgG IgA- and IgA response -in breastmilk-) in placental cells, fetal tissues, breast milk, amniotic fluid, cord blood, vaginal fluids, neonatal throat swabs. Measure also the time from birth to illness.
3. Adverse events following immunization:
  a. Maternal adverse events following immunization ^[26]^ not directly related to pregnancy outcomes (including reactogenicity).
  Late/delayed adverse event in a child believed to be linked to COVID-19 vaccination during pregnancy.
4. Immunogenicity measurements
  a. Durability of antibody response.
5. Economic outcomes: Resource use, direct and indirect costs, budget impact, and cost-effectiveness.

### 2.7. Data extraction and management

#### 1.2.7. Selection.

Pairs of review authors will independently screen each title and abstract. We will retrieve all potentially relevant full text studies and reports. Pairs of review authors will independently select the full texts, documenting the exclusion reasons for the ineligible studies.

We will resolve disagreements through discussion with the review team. This process will be performed using the web-based software COVIDENCE.^[27]^

#### 2.2.7. Data collection.

Study data will be collected and stored using REDCap electronic data capture tools^[28]^ hosted at the Institute for Clinical Effectiveness and Health Policy data servers in Buenos Aires, Argentina. Extraction forms were designed for this study, considering the wide outcome diversity to explore. Each REDCap study ID will include a general form where the principal characteristics of the studies will be included, and outcome specific forms will be generated to extract data to independently assess each endpoint reported in the studies for every outcome. The data extraction will be piloted on a sample of at least ten studies before its formal start up.

Pairs of review authors will independently extract data from included studies in a REDCap form and resolve disagreements through a discussion with the review team. If needed, we will contact the study authors by e-mail to specify any missing data which may not be reported sufficiently in the publication. Funding source information will be sought for every study included in the LSR.

Data extraction items will include study identification elements, methods, participants’ characteristics, countries involved, group allocation, intervention, outcomes, risk of bias, and summary of results.

We will use the Cochrane risk of bias tool- version 2 as recommended in the Cochrane Handbook for Systematic Reviews of Interventions.^[29]^ Each study will be evaluated regarding the following bias domains: randomization process, deviation from intended interventions, missing outcome data, measurement of outcomes, selective reporting of results, and overall assessment of the risk of bias.

For controlled before-after studies (CBAs), we will use the following criteria: Baseline measurement; characteristics for studies using the second site as a control; blinded assessment of primary outcomes; reliable measurement of primary outcomes; follow up of professionals (protection against exclusion bias); and follow up of patients. For uncontrolled before-after studies, the same criteria will be used as for CBAs, except for baseline measurement and characteristics for studies using the second site as a control. For interrupted time series, we will assess the risk of bias related to the following 7 areas: Intervention independent of other changes; shape of intervention effect predetermined; intervention unlikely to affect data collection; blinding of outcome assessors regarding intervention assignment; incomplete outcome data; selective outcome reporting; and other sources of bias. As with CBAs, we will include 3 additional domains for controlled interrupted time series trials to assess design-specific threats to validity: imbalance of outcome measures at baseline, comparability of intervention and control group characteristics at baseline, and protection from contamination.^[30]^

For observational cohort, case-control, cross-sectional and case-series studies, we will use the NIH Quality Assessment Tools. After answering the different signaling questions- Yes, No, cannot determine, not applicable, or not reported- the reviewers will classify the study quality as good, fair, or poor.^[31]^ For consistency with the other designs, we will use the classifications low, high, or unclear risk of bias, respectively.

### 2.8. Data synthesis plan

If data are available and methodologically appropriate, we will undertake the aggregate meta-analyses for each comparison according to the Cochrane Handbook of Systematic Reviews of Interventions and use the random-effects meta-analysis for the primary analysis.^[32]^ We will also perform proportion meta-analyses to summarize frequencies from 1-sample studies.

We will use R statistical software^[33]^ to analyze the data. The main packages selected for data analyses will be Meta,^[34]^ Metafor,^[35]^ and Tidyverse.^[36]^

We will calculate hazard ratios, risk ratios, or odds ratios with 95% confidence interval (95% CI) for dichotomous outcomes and mean difference or Standardized mean difference for continuous outcomes. We will also calculate proportions with 95% CI for noncomparative studies.

To report efficacy/effectiveness outcomes, we will transform other outcome measures into vaccine efficacy/effectiveness whenever possible by calculating the risk of disease among vaccinated and unvaccinated persons and determining the percentage reduction in risk of disease among vaccinated persons relative to unvaccinated persons.^[37]^

We will use adjusted effect measures (e.g., by age, smoking status, parity, body mass index, etc) over unadjusted estimates. We will investigate heterogeneity through subgroup analyses.

In the Summary of findings tables, we will summarize the grading of recommendations assessment, development, and evaluation certainty of evidence from comparative studies.^[21,38]^ The estimates will be downgraded for serious and very serious imprecision if 95% CIs crossed the null effect and the limits were < 0.95 and/or > 1.05, and < 0.5 and/or > 1.50, respectively, and for serious and very serious inconsistency if *I*^2^ values were > 60% and > 75%, respectively.

Trials with a factorial design will be included. In case of, for example, a 2 × 2 factorial design trial, the 2 groups receiving COVID-19 vaccination will be considered experimental groups, while the 2 groups receiving a placebo, “active placebo,” standard care, no intervention, or “active” comparator will be considered control groups.

We will evaluate publication bias through funnel plots when at least 8 studies are available for a specific outcome.

#### 1.2.8. Subgroup analyses.

We will perform the following prespecified subgroup analyses when analyzing the primary outcomes:

• Pregnancy trimester (first/early pregnancy 0–12 weeks; second trimester 12–27 weeks or third trimester/late pregnancy 27 weeks to full term).
• Country income level (high or low and middle-income country).
• Region (based on the Institute for Health Metrics and Evaluation categorization).
• Maternal age.
• Maternal risk status (low or high, defined as.)
• COVID-19 vaccine administered.
• Vaccine platform (mRNA, viral vector, Protein/subunit).
• Dominant variant of SARS-CoV-2 among the study population.
• Study design.
• Primary series (complete and uncompleted)/booster vaccine.

### 2.9. Sensitivity analyses

We will undertake sensitivity analyses by excluding high risk of bias studies.

### 2.10. Data visualization

We will develop an online interactive dashboard for data visualization using Microsoft Power BI. The most relevant variables will be selected among maternal and neonatal safety outcomes and presented in figures and tables. Data visualization will be delivered by primary series (complete and uncompleted)/booster vaccine.

As this project is an LSR, the living meta-analysis section will be available for users as an interactive tool developed as a Shiny application through R Studio.^[39]^ The application will allow the users to display meta-analyses of interest by selecting filters such as trimester, vaccine platform, vaccine doses, comparator, and population, among others. Predefined subgroup analyses will also be available to be selected by the users. The research team will design an algorithm for the endpoint selection of each study that will be included in the living meta-analysis. The researchers will perform a validation process to ensure the validity of the endpoint selection algorithm.

### 2.11. Ethics and dissemination

Ethical approval is not required for this study, given that this is a protocol for a systematic review and meta-analysis, which uses published data. The systematic review and living meta-analysis results will be widely disseminated via the online dashboards described above. One or more summary papers will be submitted to a leading peer review journal in this field, adhering to the Tailored PRISMA 2020 flow diagrams for living systematic reviews.^[22,23]^ When presenting our findings from this study in the Summary of findings tables, we will apply the GRADE approach for evidence from comparative studies.^[21,38]^

## Author contributions

**Conceptualization:** Agustin Ciapponi, Mabel Berrueta, Jamile Ballivian, Ariel Bardach, Agustina Mazzoni, Steven Anderson, Fernando J. Argento, Karin Bok, Daniel Comandé, Erin Goucher, Beate Kampmann, Edward P.K. Parker, Federico Rodriguez Cairoli, Victoria Santa María, Andy Stergachis, Gerald Voss, Xu Xiong, Sabra Zaraa, Flor M. Munoz, Ruth A. Karron, Sami L. Gottlieb, Pierre M. Buekens.

## Supplementary Material




