# Supplementary material for: Safety, immunogenicity, and effectiveness of COVID-19 vaccines for pregnant persons: A protocol for systematic review and meta analysis
Source: Medicine (Baltimore). 2023 Mar 3;102(9):e32954. doi: 10.1097/MD.0000000000032954 (PMC9981247; doi:10.1097/MD.0000000000032954)
Supplement: Supplementary file 2 [file medi-102-e32954-s002.pdf]

Supplementary material 1. Search strategies

PubMed search strategy

(Pregnancy[Mesh] OR Pregnan*[tiab] OR Pregnancy Complications[Mesh] OR Abortion, Spontaneous[Mesh] OR Abortion*[tiab] OR Miscarriage*[tiab] OR Gestational[tiab] OR Parturition[Mesh] OR Childbirth*[tiab] OR Parturition*[tiab] OR Partum[tiab] OR Fetus[Mesh] OR Fetal[tiab] OR Fetus[tiab] OR Maternofetal[tiab] OR Materno Fetal[tiab] OR Fetomaternal[tiab] OR Feto Maternal[tiab] OR DART[tiab]) AND (Vaccination[Mesh] OR Vaccines[Mesh] OR Vaccin*[tiab] OR Immunogenicity, Vaccine[Mesh] OR Antigenicit*[tiab] OR Immun*[tiab]) AND (COVID-19 Vaccines[Mesh] OR COVID-19 Vaccine*[tiab] OR SARS-CoV-2 Vaccine*[tiab] OR mRNA COVID-19[tiab] OR Pfizer[tiab] OR BioNTech[tiab] OR COMIRNATY[tiab] OR Tozinameran[tiab] OR AstraZeneca[tiab] OR Vaxzevria[tiab] OR AZD1222[tiab] OR Janssen[tiab] OR Covishield[tiab] OR ChAdOx1*[tiab] OR Ad26.COV2.S[tiab] OR Moderna[tiab] OR mRNA-1273[tiab] OR BIBP*[tiab] OR CoronaVac[tiab] OR Covaxin[tiab] OR NVX-CoV2373[tiab] OR Covovax[tiab] OR Novavax[tiab] OR Nuvaxovid[tiab] OR Sputnik[tiab] OR Sinovac[tiab] OR Sinopharm[tiab] OR Vero Cell[tiab] OR CanSino*[tiab] OR Ad5-nCoV[tiab] OR CoV2 preS[tiab] OR dTM-AS03[tiab] OR SCB-2019[tiab] OR CHO Cell[tiab] OR Zorecimeran[tiab] OR EpiVacCorona[tiab] OR BioCuba[tiab] OR Soberana[tiab] OR Abdala[tiab] OR Covid-19 aAPC[tiab] OR "Ad26.COV2.S"[tiab] OR BNT162*[tiab] OR aAPC Vaccine[tiab] OR Adenoviral Vector[tiab])

Filters: from 2020/1/1

(MH Pregnancy OR Pregnan$ OR Embaraz$ OR Gravid$ OR MH Pregnancy Complications OR MH Abortion, Spontaneous OR Abort$ OR Miscarriage$ OR Gestational OR Gestacional OR MH Parturition OR Parto OR Nacimiento$ OR Childbirth$ OR Parturition$ OR Partum OR MH Fetus OR Fetal OR Fetus OR Materno$ OR Fetomaternal OR Feto OR DART) AND (MH Vaccination OR MH Vaccines OR Vaccin$ OR Vacuna$ OR Vacina$ OR MH Immunogenicity, Vaccine OR

Antigenicit$ OR Immun$ OR Inmuni$ OR Antigeni$) AND (MH COVID-19 Vaccines OR ((COVID-19 OR SARS-CoV-2 OR mRNA OR aAPC) AND (Vacuna$ OR Vacina$ OR Vaccin$)) OR Pfizer OR BioNTech OR COMIRNATY OR Tozinameran OR AstraZeneca OR Vaxzevria OR AZD1222 OR Janssen OR Covishield OR ChAdOx1$ OR Ad26.COV2.S OR Moderna OR mRNA-1273 OR BIBP$ OR CoronaVac OR Covaxin OR NVX-CoV2373 OR Covovax OR Novavax OR Nuvaxovid OR Sputnik OR Sinovac OR Sinopharm OR Vero-Cell OR CanSino$ OR Ad5-nCoV OR CoV2-preS OR dTM-AS03 OR SCB-2019 OR CHO-Cell OR Zorecimeran OR EpiVacCorona OR BioCuba OR Soberana OR Abdala OR Ad26.COV2.S OR BNT162$ OR Adenoviral)

(Pregnan$ OR Abortion$ OR Miscarriage$ OR Gestational OR Childbirth$ OR Parturition$ OR Partum OR Fetal OR Fetus OR Maternofetal OR Materno Fetal OR Fetomaternal OR Feto Maternal OR DART) AND (Vaccin$ OR Antigenicit$ OR Immun$) AND (COVID-19 Vaccine$ OR SARS-CoV-2 Vaccine$ OR mRNA COVID-19 OR Pfizer OR BioNTech OR COMIRNATY OR Tozinameran OR AstraZeneca OR Vaxzevria OR AZD1222 OR Janssen OR Covishield OR ChAdOx1$ OR Ad26.COV2.S OR Moderna OR mRNA-1273 OR BIBP$ OR CoronaVac OR Covaxin OR NVX-CoV2373 OR Covovax OR Novavax OR Nuvaxovid OR Sputnik OR Sinovac OR Sinopharm OR Vero Cell OR CanSino$ OR Ad5-nCoV OR CoV2 preS OR dTM-AS03 OR SCB-2019 OR CHO Cell OR Zorecimeran OR EpiVacCorona OR BioCuba OR Soberana OR Abdala OR Covid-19 aAPC OR Ad26.COV2.S OR BNT162$ OR aAPC Vaccine OR Adenoviral Vector)

(SU=Pregnancy OR TI=Pregnan$ OR AB=Pregnan$ OR SU=Abortion OR TI=Abort$ OR AB=Abort$ OR TI=Miscarriage OR AB=Miscarriage OR TI=Gestational OR AB=Gestational OR SU=Parturition OR TI=Childbirth OR AB=Chilbirth OR TI=Parturition OR AB=Parturition OR TI=Partum OR AB=Partum OR SU=Fetus OR TI=Fetus OR AB=Fetus OR TI=Maternofetal OR AB=Maternofetal OR TI=Fetomaternal OR AB=Fetomaternal OR TI Feto Maternal OR AB=Feto Maternal OR TI=DART OR AB=DART) AND (SU=Vaccination OR TI=Vaccin$ OR AB=Vaccin$ OR SU Vaccines OR TI=Antigenicit$ OR AB=Antigenicit$ OR TI=Immun$ OR AB=Immun$) AND (SU=COVID-19 Vaccines OR TI=COVID-19 Vaccine$ OR AB=COVID-19 Vaccine$ OR TI=SARS-CoV-2 Vaccine$ OR AB=SARS-CoV-2 Vaccin$ OR TI=mRNA

COVID-19 OR AB=mRNA COVID-19 OR TI=Pfizer OR AB=Pfizer OR TI=BioNTech OR AB=BioNTech OR TI=COMIRNATY OR AB=Comirnaty OR TI=Tozinameran OR AB=Tozinameran OR TI=AstraZeneca OR AB=AstraZeneca OR TI=Vaxzevria OR AB=Vaxzevria OR TI=AZD1222 OR AB=AZD1222 OR TI=Janssen OR AB=Janssen OR TI=Covishield OR AB=Covishield OR TI=ChAdOx1$ OR AB= ChAdOx1$ OR TI=Ad26.COV2.S OR AB= Ad26.COV2.S OR TI=Moderna OR AB=Moderna OR TI=mRNA-1273 OR AB= mRNA-1273 OR TI=BIBP$ OR AB=BIBP$ OR TI=CoronaVac OR AB=CoronaVac OR TI=Covaxin OR AB=Covaxin OR TI=NVX-CoV2373 OR AB= NVX-CoV2373 OR TI=Covovax OR AB=Covovax OR TI=Novavax OR AB=Novavax OR TI=Nuvaxovid OR AB= Nuvaxovid OR TI=Sputnik OR AB=Sputnik OR TI=Sinovac OR AB=Sinovac OR TI=Sinopharm OR AB=Sinopharm OR TI=Vero Cell OR AB=Vero Cell OR TI=CanSino$ OR AB=CanSino OR TI=Ad5-nCoV OR AB= Ad5-nCoV OR TI=CoV2 preS OR AB= CoV2 preS OR TI=dTM-AS03 OR AB= dTM-AS03 OR TI=SCB-2019 OR AB= SCB-2019 OR TI=CHO Cell OR AB=CHO Cell OR TI=Zorecimeran OR AB= Zorecimeran OR TI=EpiVacCorona OR AB= EpiVacCorona OR TI=BioCuba OR AB=BioCuba OR TI=Soberana OR AB=Soberana OR TI=Abdala OR AB=Abdala OR TI=Covid-19 aAPC OR AB=Covid-19 aAPC OR TI=Ad26.COV2.S OR AB= Ad26.COV2.S OR TI=BNT162$ OR AB=BNT162$ OR TI=aAPC Vaccine OR AB= aAPC Vaccine OR TI=Adenoviral Vector OR AB=Adenoviral Vector)

(Fetal OR Fetus OR Maternofetal OR Materno Fetal OR Fetomaternal OR Feto Maternal OR DART) AND (Vaccin* OR Antigenicit* OR Immun*) AND (COVID-19 Vaccine* OR SARS-CoV-2 Vaccine* OR mRNA COVID-19 OR Pfizer OR BioNTech OR COMIRNATY OR Tozinameran OR AstraZeneca OR Vaxzevria OR AZD1222 OR Janssen OR Covishield OR ChAdOx1* OR Ad26.COV2.S OR Moderna OR mRNA-1273 OR BIBP* OR CoronaVac OR Covaxin OR NVX-CoV2373 OR Covovax OR Novavax OR Nuvaxovid OR Sputnik OR Sinovac OR Sinopharm OR Vero Cell OR CanSino* OR Ad5-nCoV OR CoV2 preS OR dTM-AS03 OR SCB-2019 OR CHO Cell OR Zorecimeran OR EpiVacCorona OR BioCuba OR Soberana OR

Abdala OR Covid-19 aAPC OR Ad26.COV2.S OR BNT162* OR aAPC Vaccine OR Adenoviral Vector)

LILACS search strategy

(MH Pregnancy OR Pregnan$ OR Embaraz$ OR Gravid$ OR MH Pregnancy Complications OR MH Abortion, Spontaneous OR Abort$ OR Miscarriage$ OR Gestational OR Gestacional OR MH Parturition OR Parto OR Nacimiento$ OR Childbirth$ OR Parturition$ OR Partum OR MH Fetus OR Fetal OR Fetus OR Materno$ OR Fetomaternal OR Feto OR DART) AND (MH Vaccination OR MH Vaccines OR Vaccin$ OR Vacuna$ OR Vacina$ OR MH Immunogenicity, Vaccine OR Antigenicit$ OR Immun$ OR Inmuni$ OR Antigeni$) AND (MH COVID-19 Vaccines OR ((COVID-19 OR SARS-CoV-2 OR mRNA OR aAPC) AND (Vacuna$ OR Vacina$ OR Vaccin$)) OR Pfizer OR BioNTech OR COMIRNATY OR Tozinameran OR AstraZeneca OR Vaxzevria OR AZD1222 OR Janssen OR Covishield OR ChAdOx1$ OR Ad26.COV2.S OR Moderna OR mRNA-1273 OR BIBP$ OR CoronaVac OR Covaxin OR NVX-CoV2373 OR Covovax OR Novavax OR Nuvaxovid OR Sputnik OR Sinovac OR Sinopharm OR Vero-Cell OR CanSino$ OR Ad5-nCoV OR CoV2-preS OR dTM-AS03 OR SCB-2019 OR CHO-Cell OR Zorecimeran OR EpiVacCorona OR BioCuba OR Soberana OR Abdala OR Ad26.COV2.S OR BNT162$ OR Adenoviral) [Words] and 2020 OR 2021 OR 2022 [Country, year publication]

CNKI search strategy

(SU=Pregnancy OR TI=Pregnan$ OR AB=Pregnan$ OR SU=Abortion OR TI=Abort$ OR AB=Abort$ OR TI=Miscarriage OR AB=Miscarriage OR TI=Gestational OR AB=Gestational OR SU=Parturition OR TI=Childbirth OR AB=Chilbirth OR TI=Parturition OR AB=Parturition OR TI=Partum OR AB=Partum OR SU=Fetus OR TI=Fetus OR AB=Fetus OR TI=Maternofetal OR AB=Maternofetal OR TI=Fetomaternal OR AB=Fetomaternal OR TI Feto Maternal OR AB=Feto Maternal OR TI=DART OR AB=DART) AND (SU=Vaccination OR TI=Vaccin$ OR

AB=Vaccin$ OR SU Vaccines OR TI=Antigenicit$ OR AB=Antigenicit$ OR TI=Immun$ OR AB=Immun$) AND (SU=COVID-19 Vaccines OR TI=COVID-19 Vaccine$ OR AB=COVID-19 Vaccine$ OR TI=SARS-CoV-2 Vaccine$ OR AB=SARS-CoV-2 Vaccin$ OR TI=mRNA COVID-19 OR AB=mRNA COVID-19 OR TI=Pfizer OR AB=Pfizer OR TI=BioNTech OR AB=BioNTech OR TI=COMIRNATY OR AB=Comirnaty OR TI=Tozinameran OR AB=Tozinameran OR TI=AstraZeneca OR AB=AstraZeneca OR TI=Vaxzevria OR AB=Vaxzevria OR TI=AZD1222 OR AB=AZD1222 OR TI=Janssen OR AB=Janssen OR TI=Covishield OR AB=Covishield OR TI=ChAdOx1$ OR AB= ChAdOx1$ OR TI=Ad26.COV2.S OR AB= Ad26.COV2.S OR TI=Moderna OR AB=Moderna OR TI=mRNA-1273 OR AB= mRNA-1273 OR TI=BIBP$ OR AB=BIBP$ OR TI=CoronaVac OR AB=CoronaVac OR TI=Covaxin OR AB=Covaxin OR TI=NVX-CoV2373 OR AB= NVX-CoV2373 OR TI=Covovax OR AB=Covovax OR TI=Novavax OR AB=Novavax OR TI=Nuvaxovid OR AB= Nuvaxovid OR TI=Sputnik OR AB=Sputnik OR TI=Sinovac OR AB=Sinovac OR TI=Sinopharm OR AB=Sinopharm OR TI=Vero Cell OR AB=Vero Cell OR TI=CanSino$ OR AB=CanSino OR TI=Ad5-nCoV OR AB= Ad5-nCoV OR TI=CoV2 preS OR AB= CoV2 preS OR TI=dTM-AS03 OR AB= dTM-AS03 OR TI=SCB-2019 OR AB= SCB-2019 OR TI=CHO Cell OR AB=CHO Cell OR TI=Zorecimeran OR AB= Zorecimeran OR TI=EpiVacCorona OR AB= EpiVacCorona OR TI=BioCuba OR AB=BioCuba OR TI=Soberana OR AB=Soberana OR TI=Abdala OR AB=Abdala OR TI=Covid-19 aAPC OR AB=Covid-19 aAPC OR TI=Ad26.COV2.S OR AB= Ad26.COV2.S OR TI=BNT162$ OR AB=BNT162$ OR TI=aAPC Vaccine OR AB= aAPC Vaccine)

COVID-19 Global literature on coronavirus disease (WHO)

(Pregnan$ OR Abortion$ OR Miscarriage$ OR Gestational OR Childbirth$ OR Parturition$ OR Partum OR Fetal OR Fetus OR Maternofetal OR Materno Fetal OR Fetomaternal OR Feto Maternal OR DART) AND (Vaccin$ OR Antigenicit$ OR Immun$) AND (COVID-19 Vaccine$ OR SARS-CoV-2 Vaccine$ OR mRNA COVID-19 OR Pfizer OR BioNTech OR COMIRNATY OR Tozinameran OR AstraZeneca OR Vaxzevria OR AZD1222 OR Janssen OR Covishield OR

ChAdOx1$ OR Ad26.COV2.S OR Moderna OR mRNA-1273 OR BIBP$ OR CoronaVac OR Covaxin OR NVX-CoV2373 OR Covovax OR Novavax OR Nuvaxovid OR Sputnik OR Sinovac OR Sinopharm OR Vero Cell OR CanSino$ OR Ad5-nCoV OR CoV2 preS OR dTM-AS03 OR SCB-2019 OR CHO Cell OR Zorecimeran OR EpiVacCorona OR BioCuba OR Soberana OR Abdala OR Covid-19 aAPC OR Ad26.COV2.S OR BNT162$ OR aAPC Vaccine OR Adenoviral Vector)

L-OVE Database search strategy

(Pregnan* OR Abortion* OR Miscarriage* OR Gestational OR Childbirth* OR Parturition* OR Partum OR Fetal OR Fetus OR Maternofetal OR Materno Fetal OR Fetomaternal OR Feto Maternal OR DART) AND (Vaccin* OR Antigenicit* OR Immun*) AND (COVID-19 Vaccine* OR SARS-CoV-2 Vaccine* OR mRNA COVID-19 OR Pfizer OR BioNTech OR COMIRNATY OR Tozinameran OR AstraZeneca OR Vaxzevria OR AZD1222 OR Janssen OR Covishield OR ChAdOx1* OR Ad26.COV2.S OR Moderna OR mRNA-1273 OR BIBP* OR CoronaVac OR Covaxin OR NVX-CoV2373 OR Covovax OR Novavax OR Nuvaxovid OR Sputnik OR Sinovac OR Sinopharm OR Vero Cell OR CanSino* OR Ad5-nCoV OR CoV2 preS OR dTM-AS03 OR SCB-2019 OR CHO Cell OR Zorecimeran OR EpiVacCorona OR BioCuba OR Soberana OR Abdala OR Covid-19 aAPC OR Ad26.COV2.S OR BNT162* OR aAPC Vaccine OR Adenoviral Vector)

MedRxiv and bioRxiv search strategy

"Pregnancy AND Vaccine AND Covid" and posted between "01 Jan, 2020 and 15 Feb, 2022"
